# Risk Heterogeneity of Liver-Related Events and Extrahepatic Outcomes Across MASLD Phenotypes and Risk Stratification by Liver Fibrosis

**DOI:** 10.1155/ije/1262001

**Published:** 2025-06-01

**Authors:** Xue Bao, Xiaowen Zhang, Dahui Xu, Yu Wang, Songjiang Yin, Xinlin Zhang

**Affiliations:** ^1^Department of Cardiology, Nanjing Drum Tower Hospital, Affiliated Hospital of Medical School, Nanjing University, Nanjing, Jiangsu, China; ^2^Endocrine and Metabolic Disease Medical Center, Nanjing Drum Tower Hospital, Affiliated Hospital of Medical School, Nanjing University, Nanjing, Jiangsu, China; ^3^Department of Biliopancreatic Surgery, Nankai Hospital, Tianjin, China; ^4^First College of Clinical Medicine, Nanjing University of Chinese Medicine, Nanjing, Jiangsu, China

**Keywords:** cancer, MASLD, prospective, United Kingdom's Biobank

## Abstract

**Background:** Metabolic dysfunction–associated steatotic liver disease (MASLD) has been newly proposed to characterize fatty liver disease. We aim to investigate the associations of different phenotypes of MASLD and related steatotic liver disease (SLD) with the risk of liver-related and extrahepatic outcomes.

**Methods:** Among 368,886 United Kingdom's Biobank participants, those with MASLD and related SLD were categorized into pure MASLD, MASLD with increased alcohol intake (MetALD), MASLD with alcohol-related liver disease (ALD), and MASLD with other etiology. The primary outcome was liver-related events (LREs).

**Results:** During a median follow-up of 13.7 years, 2095 participants developed LREs. The adjusted HRs (95% CIs) of LREs for pure MASLD, MetALD, MASLD with ALD, and MASLD with other etiology were 2.46 (2.21, 2.73), 2.77 (2.39, 3.21), 8.73 (7.59, 10.1), and 26.5 (17.1, 41.0), respectively. Participants with MetALD, MASLD with ALD, and MASLD with other etiology showed a considerably higher risk of liver-related outcomes but a modestly higher risk of extrahepatic cancer compared to those with pure MASLD. A remarkably higher risk of LREs was observed in participants with a fibrosis-4 > 2.67.

**Conclusion:** MASLD and related SLD are associated with increased risks of LREs and extrahepatic outcomes, with heterogeneous risks across different phenotypes and significant risk stratification by liver fibrosis severity.

## 1. Introduction

Nonalcoholic fatty liver disease (NAFLD) affects approximately one-quarter of the global population [1]. Recent evidence suggests that NAFLD is not solely a liver-related disease but rather a complex condition with significant extrahepatic implications, including cardiovascular disease (CVD) and various cancers [2, 3]. However, the exclusion of alcohol in the diagnosis of NAFLD has been a contentious issue, as it fails to accurately capture the disease's true origins and may lead to stigma and confusion.

To address these concerns, an international panel of experts proposed clear criteria for diagnosing metabolic dysfunction–associated fatty liver disease (MAFLD), shifting the diagnostic approach from exclusion to inclusion [4, 5]. Despite this, MAFLD has not been universally accepted due to concerns about mixed etiologies and potential exclusions of lean and normal-weight individuals with NAFLD [6].

In 2023, major liver disease (LD) associations introduced a new nomenclature, metabolic dysfunction–associated steatotic liver disease (MASLD), to characterize fatty LD [7]. This consensus includes separate disease entities such as MASLD with increased alcohol intake (MetALD) and MASLD with other combined etiology, acknowledging varying levels of alcohol consumption and avoiding complex criteria such as insulin resistance [7].

While some recent publications have highlighted the association between MASLD and increased cardiovascular risk and mortality [8, 9], the transition from NAFLD to an etiology and inclusion-based definition of MASLD has not been extensively investigated. No study to date has comprehensively assessed the relationship between MASLD and liver-related events (LREs) and extrahepatic cancers in the general population. In addition, the predictive value of the fibrosis-4 (FIB-4) index, a widely used tool for predicting LREs in NAFLD [10], in the context of MASLD and related steatotic LD (SLD) remains uncertain. Therefore, our study aims to explore the independent longitudinal association of MASLD and related SLD with LREs and extrahepatic cancers. Furthermore, we seek to determine the predictive effectiveness of the FIB-4 index in differentiating LREs in individuals with these disease entities.

## 2. Materials and Methods

### 2.1. Participants

**Figure 1:**
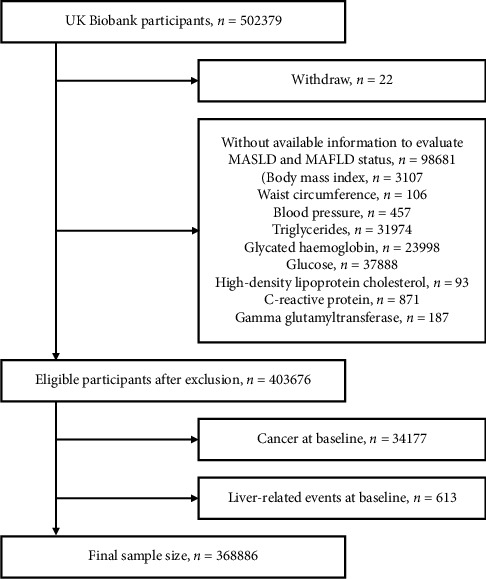
Study population flowchart.

The United Kingdom's Biobank is a nationwide prospective cohort study with a sample size of over 500,000. The participants were recruited between 2006 and 2010 at one of the 22 assessment centers located across England, Scotland, and Wales, and ranged in age from 38 to 72 years [11]. This project was conducted under application number 91907. After excluding 22 withdrawn participants, there were 403,676 participants with complete data eligible for evaluating MASLD and MAFLD status. We then excluded those with pre-existing LREs and cancer, except for certain types of skin cancer, based on registry diagnoses (Supporting Information: Table S1). Self-reported cirrhosis and cancer except for nonmelanoma skin cancer were also excluded. As a result, 368,886 participants remained for analysis (Figure 1). Ethical approval for the study was obtained from the North West Multicenter Research Ethics Committee, and written informed consent was obtained from all participants. The study adhered to the STROBE guidelines and was conducted according to the Declaration of Helsinki.

### 2.2. MASLD and Related SLD

Hepatic steatosis was defined by using a previously validated score, the fatty liver index (FLI). The FLI has demonstrated reliability as an alternative to imaging techniques such as ultrasonography and transient elastography, showing a good diagnostic performance with an area under the receiver operator curve of 0.85 [12]. A FLI < 30 rules out and a FLI ≥ 60 rules in hepatic steatosis as detected by ultrasonography [13]. In the main analyses, MASLD was defined as FLI ≥ 60 with ≥ 1 predefined cardiometabolic risk factor, such as overweight or abnormalities in glucose, blood pressure, lipids, or high-density lipoprotein cholesterol [14]. Those with increased or excessive alcohol intake (weekly intake ≥ 210 g for males or ≥ 140 g for females) or concomitant LD were specifically excluded from pure MASLD [14]. MetALD was defined as SLD and ≥ 1 cardiometabolic risk factor, along with an increased alcohol intake of 210–420 g per week for males or 140–350 g for females. Those with excessive alcohol intake (weekly intake > 420 g for males or > 350 g for females) or alcohol-related LD (ALD), in addition to SLD and ≥ 1 cardiometabolic risk factor, were considered to have MASLD with ALD. MASLD with other etiology was defined as SLD with ≥ 1 cardiometabolic risk factor and concomitant LDs. Consistent with previous definitions, pure MASLD, MetALD, MASLD with ALD, and MASLD with other etiology were collectively referred to as “MASLD and related SLD” [9]. A very small group of participants (*n* = 41) had both excessive alcohol consumption or ALD and other concomitant LDs. To avoid overlapping, they were excluded from “MASLD with ALD” and “MASLD with other etiology.” MAFLD was defined as SLD with either overweight or obesity, Type 2 diabetes, or at least two metabolic abnormalities, and additional analyses were conducted with participants categorized by MAFLD status [15].

### 2.3. Assessment of Covariates

Race was classified into five categories: White, Asian, Black, Mixed, or other. Education was categorized as either holding a college or university degree or not. Smoking status was classified as current, former, or never smokers. Alcohol intake was categorized as daily or almost daily, 3-4 times per week, 1-2 times per week, occasionally, or never. The Townsend deprivation index (TDI), a measure of socioeconomic status, was divided into quartiles, with higher scores indicating lower socioeconomic status. Annual household income was categorized as <£18,000, £18,000–30999, £31,000–51999, or >£52,000. Self-reported physical activity was categorized as high, moderate, or low, based on the validated International Physical Activity Questionnaire (IPAQ). CVDs were defined as a composite of ischemic heart disease, stroke, and heart failure. The estimated glomerular filtration rate (eGFR) was calculated using the chronic kidney disease epidemiology collaboration equation and categorized using cutoff values of 30, 60, and 90 mL/min/1.73 m^2^ [16]. The FIB-4 index was calculated as previously described [17] and categorized into three degrees: low (< 1.3), intermediate (≥ 1.3 to ≤ 2.67), and high (> 2.67).

### 2.4. Primary and Secondary Outcomes

The primary outcome was LREs, defined by International Classification of Disease-10 (ICD-10) codes specified in Supporting Information: Table S1, through linkage to hospital admission records as either primary or secondary diagnosis. For patients with multiple diagnosis codes, the earliest one was used. Secondary outcomes included liver cirrhosis and cirrhosis-related complications, cancer, hepatocellular carcinoma, extrahepatic cancer, all-cause death, liver-related death, and cancer death (Supporting Information: Table S1). The primary cause was used to identify cause-specific death. Participants were followed from enrollment until the occurrence of an outcome event, death, or 31 October 2022, whichever came first. The United Kingdom's Biobank Outcome Adjudication Group conducted outcome adjudication.

### 2.5. Statistical Analysis

Participants were categorized by the presence of the subtypes of MASLD and related SLD, and baseline characteristics were described as mean ± standard deviation for continuous variables and as numbers and percentages for categorical variables. Cox proportional hazards regression models were used to assess the associations between MASLD and related SLD with outcomes. The proportional hazards assumption was validated using Schoenfeld residuals. The models were adjusted for age, sex, ethnicity, education, TDI, income levels, smoking and drinking status, IPAQ activity levels, eGFR, and CVDs. Missing data were coded separately for categorical variables. Participants (*n* = 41) with both excessive alcohol consumption or ALD and other concomitant LDs were coded as a separate group and were also adjusted for in the model. To prevent potential overadjustment, certain cardiometabolic risk factors outlined in the MASLD criteria were not included in the main analysis [8, 18]. However, they were considered in a sensitivity analysis. Additional sensitivity analyses were also conducted. Participants with incident events during the first 2 years of follow-up were excluded to address potential reverse causality. Notably, competing risk analysis is an important consideration in high-mortality cohorts (e.g., older populations or long-term studies) where Kaplan–Meier estimates or traditional Cox regression may inflate event risk by assuming that those who died are still at risk at the point of censoring [19, 20]. To address this, we implemented the fine-gray proportional subhazards models for primary outcome analysis, treating death from other causes as a competing event. This approach employs cumulative incidence function estimation through subdistribution hazards, which appropriately accounts for the probability of the primary event occurring in the presence of competing risks [21]. A lower cutoff of FLI ≥ 30, instead of ≥ 60, is used to define SLD. Lastly, the interactions between FIB-4 categories and MASLD and related SLD were tested for their association with LREs. The associations of MAFLD with outcomes were also presented as supporting information. All statistical analyses were carried out using SAS software, Version 9.4 (SAS Institute Inc, Cary, NC). A *p* value less than 0.05 (two-tailed) was considered statistically significant.

## 3. Results

### 3.1. Baseline Characteristics

Among the 368,886 participants (aged 56.2 ± 8.11 years, 47.0% male), the prevalence of pure MASLD, MetALD, MASLD with ALD, and MASLD with other etiology was 27.9%, 7.74%, 2.87% and 0.07%, respectively. Compared to participants without MASLD or related SLD, those with overall MASLD and related SLDs tended to be older, physically inactive, and more likely to be male and smokers. They also had a higher body mass index (BMI) and lower levels of education, socioeconomic status, household income, and eGFR, as well as a higher prevalence of diabetes, hypertension, and CVDs (data not shown). Baseline characteristics of participants without MASLD or related SLD, as well as those for each subtype of MASLD and related SLD, are presented in Table 1.

### 3.2. Association of MASLD With LREs and Other Outcomes

During a median follow-up of 13.7 years (interquartile range: 12.9–14.4 years), 2095 participants developed LREs. After multivariate adjustment, as compared to participants without MASLD or related SLD, all subtypes of MASLD and related SLD were associated with a significantly higher risk of LREs. The HRs (95% CIs) were 2.46 (2.21, 2.73) for pure MASLD, 2.77 (2.39, 3.21) for MetALD, 8.73 (7.59, 10.1) for MASLD with ALD, and 26.5 (17.1, 41.0) for MASLD with other etiology (all *p* < 0.0001) (Table 2). An increased risk of all hepatic outcomes was observed in each subgroup of MASLD and related SLD (all *p* < 0.0001) (Table 2). For liver cirrhosis and cirrhosis-related complications, the HRs (95% CIs) were 2.59 (2.31, 2.89) for pure MASLD, 2.90 (2.49, 3.38) for MetALD, 9.54 (8.26, 11.0) for MASLD with ALD, and 23.8 (14.6, 38.6) for MASLD with other etiologies (all *p* < 0.0001). For hepatocellular carcinoma (HCC), the HRs (95% CIs) were 2.01 (1.50, 2.69) for pure MASLD, 3.51 (2.36, 5.21) for MetALD, 7.19 (4.67, 11.1) for MASLD with ALD, and 76.3 (38.1, 152.8) for MASLD with other etiologies (all *p* < 0.0001). For liver-related death, the HRs (95% CIs) were 1.99 (1.67, 2.38) for pure MASLD, 3.06 (2.43, 3.84) for MetALD, 8.92 (7.12, 11.2) for MASLD with ALD, and 29.6 (15.2, 57.7) for MASLD with other etiologies (all *p* < 0.0001). Participants with MetALD, MASLD with ALD, and MASLD with other etiologies exhibited a considerably higher risk of liver-related outcomes compared to those with pure MASLD.

In contrast, all subgroups of MASLD and related SLD were associated with a mild-to-moderate increase in the risk of extrahepatic outcomes (Table 2). The HRs for cancer, extrahepatic carcinoma, all-cause death, and cancer death ranged from 1.12 to 1.32, with relatively higher risks observed in the subgroup of MASLD with other etiologies (HRs ranging from 1.51 to 3.73).

### 3.3. Association of MAFLD With LREs and Other Outcomes

The association of MAFLD with outcomes is presented in Supporting Information: Table S2. The risk for LREs and other outcomes comparing participants with and without MAFLD was comparable to that observed in participants with overall MASLD and related SLDs. The HRs (95% CIs) were 2.91 (2.64, 3.21) for LREs, 3.07 (2.78, 3.40) for liver cirrhosis and cirrhosis-related complications, 2.74 (2.11, 3.57) for HCC, and 2.59 (2.22, 3.03) for liver-related death (all *p* < 0.0001). Meanwhile, the HRs for cancer, extrahepatic carcinoma, all-cause death, and cancer death ranged from 1.12 to 1.29 (all *p* < 0.0001).

### 3.4. Subgroup and Sensitivity Analyses

Consistent results were observed when additionally adjusting for BMI, diabetes, hypertension, and hyperlipidemia (Supporting Information: Table S3) or including only participants with outcomes occurring after a follow-up duration of ≥ 2 years (Supporting Information: Table S4). Moreover, taking the competing risk of death from other causes into account or defining SLD as a FLI ≥ 30 also showed comparable findings (Supporting Information: Tables S5 and S6).

### 3.5. FIB-4 Index and Risk of LREs

**Figure 2:**
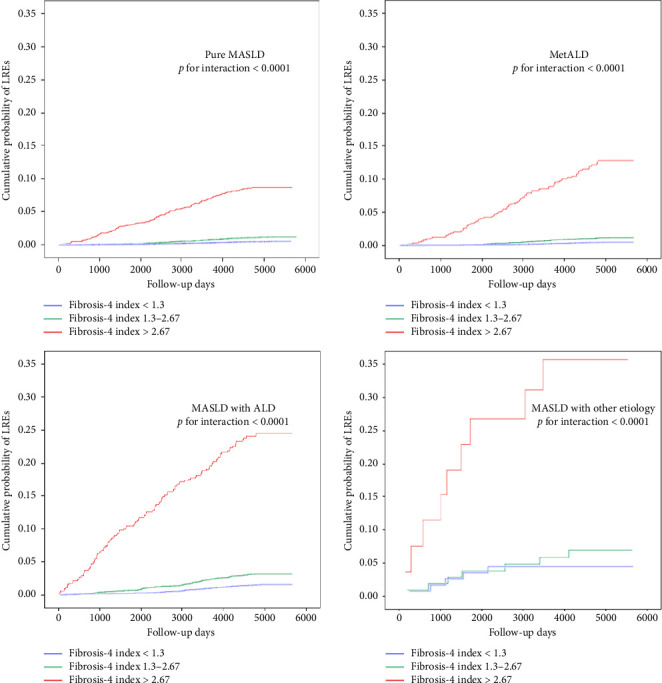
Kaplan–Meier plots of FIB-4 categories for LREs by subtypes of MASLD and related SLD. ALD: alcohol fatty liver disease; LREs: liver-related events; MASLD: metabolic dysfunction–associated steatotic liver disease; SLD, steatotic liver disease.

A significant interaction was observed across FIB-4 categories and MASLD and related SLD in terms of the risk of LREs. The risk increased with increasing FIB-4 index for each subtype of MASLD and related SLD, with a FIB-4 > 2.67 showing a remarkably higher risk of LREs.  Figure 2 displays the Kaplan–Meier plots of different categories of FIB-4 for LREs by subtypes of MASLD and related SLD.

## 4. Discussion

Approximately 38.6% of the United Kingdom's Biobank participants were classified as having MASLD and related SLD at baseline. All subtypes of MASLD and related SLD were associated with a higher risk of LREs and all-cause mortality. MetALD, MASLD with ALD, and MASLD with other etiology were associated with a higher risk of liver-related outcomes than pure MASLD. Notably, the FIB-4 index showed promising predictive efficacy in differentiating LREs in the context of MASLD and related SLD.

Our study demonstrates that MASLD and increased alcohol intake are independent predictors for LREs, and their coexistence results in an additive higher risk of LREs. Even modestly increased alcohol intake, as seen in MetALD, increases the risk of LREs, although showing a similar risk of extrahepatic outcomes compared to pure MASLD. This observation is supported by recent findings from a study involving 2535 individuals undergoing magnetic resonance imaging elastography (MRE) and proton density fat fraction in primary care settings, where significantly higher MRE and FIB-4 scores were observed in the MetALD group compared to the pure MASLD group [22]. It is suggested that modestly increased alcohol may impact clinical phenotypes and prognoses of metabolic dysfunction [23], and an excess alcohol consumption could remarkably increase the risk of LREs.

The relationship between alcohol and MASLD is complex, with evidence suggesting additive or synergistic effects on the risk of LREs between alcohol and cardiometabolic dysfunction [24]. In our analysis, MASLD with excess alcohol intake markedly increases the risk of LREs, as well as extrahepatic outcomes. ALD has consistently demonstrated a poorer long-term prognosis than NAFLD [25]. While much of this evidence originates from the NAFLD era, it supports the findings of this MASLD–oriented article [26].

It is worth noting that MASLD with excess alcohol was much more frequent than MASLD with other etiology, such as viral hepatitis and autoimmune hepatitis. It is essential to highlight that, besides alcohol use, these patients have cardiometabolic dysfunction and may not be in the same group as those with ALD without MASLD. It is interesting that the risk of LREs observed in participants with MASLD with other etiology was much higher than MASLD with alcohol consumption.

We revealed that the risk of outcomes was comparable between overall MASLD and SLDs and MAFLD. This suggests that the simplified metabolic criteria employed in the MASLD definition, without including high-sensitivity C-reactive protein and fasting insulin level criteria found in the MAFLD definition, could still identify individuals with similarly elevated LRE risk. Furthermore, the MASLD's new nomenclature allows for more effective disease stratification than MAFLD after considering the amount of alcohol consumption and other etiology, and therefore should be encouraged in clinical practice.

HCC represents the majority of primary liver cancer cases, comprising 70%–85% of cases and ranking among the most prevalent malignancies worldwide [27]. Our investigation indicates that individuals with pure MASLD face twice the risk of developing HCC over the long term, whereas those with MetALD, MASLD with ALD, and MASLD stemming from other causes experience even greater risks, ranging from 3.5 to 70 times higher. In contrast, the risk of extrahepatic cancers sees only a mild increase among the subtypes of MASLD and related SLD. These findings underscore the heightened risk of HCC associated with alcohol consumption. In contrast, similar to the report on NAFLD, the impact on extrahepatic cancers appears to be modest [2]. The precise mechanisms through which MASLD may contribute to the development of extrahepatic cancers remain elusive but are likely related to MASLD itself and the shared metabolic risk factors.

The FIB-4 index, an easily calculable clinical tool widely utilized for screening and risk assessment in NAFLD, has gained prominence in recent clinical care guidelines [28]. According to the American Gastroenterological Association's clinical care pathway, individuals with two or more metabolic risk factors, Type 2 diabetes, or evidence of steatosis on imaging or elevated aminotransferases are considered at significant risk and should undergo screening for NAFLD–related fibrosis [29]. Although the validation of the FIB-4 index in MASLD patients is currently lacking, recent evidence suggests its potential as a prognostic indicator for liver-related outcomes in NAFLD [10, 30]. In our study, we for the first time investigated the potential impact of the FIB-4 index on differentiating the risk of LREs across various groups of MASLD. In agreement with findings from NAFLD, MASLD patients with a FIB-4 index > 2.67 showed a remarkably higher risk of LREs, indicating a higher risk of liver fibrosis. It is reasonable to posit that the FIB-4 index observations derived from earlier NAFLD studies retain their relevance within the context of the updated MASLD classification irrespective of alcohol consumption, and conducting further validation studies on the FIB-4 index during the transition from NAFLD to MASLD may be deemed unnecessary. However, it is important to note that MASLD, even in individuals with a FIB-4 index < 1.3, still confers a heightened risk of LREs compared to those without MASLD or related SLD.

This study has several strengths, including its prospective design, large sample size, extensive long-term follow-up, robust adjustment for potential confounding factors, and sensitivity analyses for strengthening the results, e.g., the use of two predefined FLI cutoffs (≥ 60 for clinical relevance and ≥ 30 to account for subclinical heterogeneity), with consistent outcomes across both thresholds underscoring the reliability of our findings. However, it is important to acknowledge some limitations. First, hepatic steatosis was defined using the FLI rather than liver biopsy or imaging. However, the FLI has shown a strong correlation with ultrasound diagnosis of NAFLD in multiple studies [31] and is widely used in recent studies of MASLD [9, 32]. Our sensitivity analyses using different FLI cutoffs also showed similar findings. Second, MASLD status and alcohol consumption were assessed solely at baseline, lacking longitudinal follow-up data to capture dynamic changes over time. This static assessment may result in misclassification of exposure, potentially leading to an underestimation of true associations. However, given the robustness of our findings, it is unlikely that such misclassification would entirely negate the observed associations. Future studies incorporating repeated measurements are essential to further validate these observations. Third, despite controlling for various confounding factors, the potential for residual confounding exists. Fourth, as the findings are observational, causality cannot be established. Finally, the majority of participants in the United Kingdom's Biobank study were of White ethnicity, so generalizing the findings to other ethnic groups should be performed with caution.

## 5. Conclusions

The presence of MASLD and related SLD are associated with an increased risk of LREs, extrahepatic cancers, and liver- and cancer-related mortality. A high-risk FIB-4 index demonstrated a significant independent association with LREs within the context of MASLD and related SLD. These findings indicate that the introduction of the new MASLD nomenclature allows for more effective disease stratification than MAFLD after considering the amount of alcohol consumption and other etiology and confirmed that FIB-4 index findings from older NAFLD studies remain valid under the new MASLD definition. Our findings also underscore the importance of ongoing and future trials aimed at investigating whether the regression of MASLD and related SLD may lead to a reduction in LREs.

## Figures and Tables

**Table 1:** Baseline characteristics of study participants according to the presence of the subtypes of MASLD and related SLD.

	No MASLD/related SLD (*N* = 226,639)	Pure MASLD^a^ (*N* = 102,821)	MetALD^b^ (*N* = 28,563)	MASLD with ALD^c^ (*N* = 10,577)	MASLD with other etiology^d^ (*N* = 245)
Age (years)	55.8 ± 8.22	57.1 ± 7.92	56.9 ± 7.68	56.3 ± 7.64	57.2 ± 7.58
Sex					
Female	145,762 (64.3)	40,379 (39.3)	7425 (26)	1758 (16.6)	65 (26.5)
Male	80,877 (35.7)	62,442 (60.7)	21,138 (74)	8819 (83.4)	180 (73.5)
Body mass index (kg/m^2^)					
< 18.5	1848 (0.82)	0 (0.00)	0 (0.00)	1 (0.01)	0 (0.00)
≥ 18.5 to < 25.0	116,216 (51.3)	1932 (1.88)	796 (2.79)	477 (4.51)	5 (2.04)
≥ 25.0 to < 30.0	99,235 (43.8)	39,742 (38.7)	13,538 (47.4)	5024 (47.5)	109 (44.5)
≥ 30.0	9340 (4.12)	61,147 (59.5)	14,229 (49.8)	5075 (48)	131 (53.5)
Ethnicity					
White	214,034 (94.8)	95,635 (93.6)	27,977 (98.2)	10,353 (98.2)	222 (91.0)
Asian	5308 (2.35)	2907 (2.84)	169 (0.59)	32 (0.30)	0 (0.00)
Black	2975 (1.32)	1922 (1.88)	139 (0.49)	70 (0.66)	7 (2.87)
Mixed	1395 (0.62)	598 (0.59)	110 (0.39)	46 (0.44)	11 (4.51)
Other	1957 (0.87)	1146 (1.12)	87 (0.31)	37 (0.35)	4 (1.64)
Education					
College or university degree	81,808 (36.1)	26,050 (25.4)	8636 (30.2)	7517 (71.1)	176 (71.8)
Other	144,632 (63.9)	76,609 (74.6)	19,927 (69.8)	3059 (28.9)	69 (28.2)
Townsend deprivation index quartile					
Q1 (least deprived)	59,633 (26.3)	22,965 (22.4)	7225 (25.3)	2289 (21.7)	51 (20.8)
Q2	58,246 (25.7)	24,257 (23.6)	7189 (25.2)	2333 (22.1)	42 (17.1)
Q3	56,472 (24.9)	25,573 (24.9)	7298 (25.6)	2688 (25.4)	51 (20.8)
Q4 (most deprived)	52,021 (23.0)	29,891 (29.1)	6821 (23.9)	3252 (30.8)	101 (41.2)
Income levels					
Level 1 (< £18,000)	38,790 (20.0)	24,381 (28.1)	4559 (17.8)	2079 (21.7)	66 (32.2)
Level 2 (£8000–30999)	48,152 (24.8)	22,959 (26.4)	6151 (24.0)	2186 (22.8)	50 (24.4)
Level 3 (£31,000–52000)	52,070 (26.8)	21,594 (24.9)	7169 (28.0)	2472 (25.8)	45 (22.0)
Level 4 (> £52,000)	54,944 (28.3)	17,882 (20.6)	7716 (30.1)	2843 (29.7)	44 (21.5)
Smoking status					
Never	133,212 (59.0)	54,606 (53.5)	10,857 (38.2)	3123 (29.6)	92 (38.2)
Past	69,880 (31.0)	37,106 (36.3)	13,838 (48.6)	5145 (48.8)	106 (44.0)
Current	22,587 (10.0)	10,409 (10.2)	3759 (13.2)	2284 (21.6)	43 (17.8)
Alcohol intake					
Daily or almost daily	45,800 (20.2)	6883 (6.72)	14,020 (49.1)	8269 (78.3)	38 (15.7)
3-4 times a week	54,576 (24.1)	17,999 (17.6)	11,782 (41.2)	1583 (15.0)	42 (17.4)
1-2 times a week	59,445 (26.3)	33,075 (32.3)	2761 (9.67)	346 (3.28)	64 (26.4)
Occasionally	49,551 (21.9)	32,834 (32.0)	0 (0.00)	154 (1.46)	58 (24.0)
Never	16,832 (7.44)	11,709 (11.4)	0 (0.00)	207 (1.96)	40 (16.5)
IPAQ activity group					
Low	28,418 (15.4)	20,152 (25.1)	5196 (21.8)	2192 (24.2)	60 (30.0)
Moderate	75,431 (40.8)	32,613 (40.6)	9843 (41.3)	3524 (39.0)	79 (39.5)
High	81,250 (43.9)	27,624 (34.4)	8807 (36.9)	3327 (36.8)	61 (30.5)
Diabetes					
No	220,584 (97.3)	89,550 (87.1)	26,357 (92.3)	9613 (90.89)	194 (79.2)
Yes	6055 (2.67)	13,271 (12.9)	2206 (7.72)	964 (9.11)	51 (20.8)
Hypertension					
No	180,229 (79.5)	59,437 (57.8)	16,639 (58.3)	5541 (52.4)	113 (46.1)
Yes	46,410 (20.5)	43,384 (42.2)	11,924 (41.7)	5036 (47.6)	132 (53.9)
Cardiovascular disease					
No	215,948 (95.3)	91,815 (89.3)	26,123 (91.5)	9427 (89.1)	214 (87.4)
Yes	10,691 (4.72)	11,006 (10.7)	2440 (8.54)	1150 (10.9)	31 (12.7)
Estimated glomerular filtration rate (mL/min/1.73 m^2^)^e^					
< 30	120 (0.05)	156 (0.15)	19 (0.07)	5 (0.05)	0 (0.00)
≥ 30 to < 60	3270 (1.44)	3241 (3.15)	509 (1.78)	159 (1.50)	13 (5.31)
≥ 60 to < 90	81,287 (35.9)	43,062 (41.9)	11,109 (38.9)	3493 (33.0)	87 (35.5)
≥ 90	141,934 (62.6)	56,352 (54.8)	16,922 (59.3)	6920 (65.4)	145 (59.2)

*Note:* Age is presented as mean ± standard deviation. All other data are presented as *n* (%). Concomitant LDs include viral hepatitis (B15–B19), alcoholic liver disease (K70), toxic liver disease (K71), biliary cholangitis (K74.3–K74.5), autoimmune hepatitis (K75.4), Wilson's disease (E83.0), or hemochromatosis (E83.1); in parentheses are the International Classification of Disease-10 codes.

Abbreviations: ALD = alcohol fatty liver disease; IPAQ = International Physical Activity Questionnaire; LD = liver disease; MASLD = metabolic dysfunction–associated steatotic liver disease; SLD = steatotic liver diseases.

^a^Defined as SLD with ≥ 1 predefined cardiometabolic risk factor. Participants with increased or excessive alcohol intake (weekly intake ≥ 210 g for males or ≥ 140 g for females) or concomitant LDs were excluded.

^b^Defined as SLD with ≥ 1 cardiometabolic risk factor and an increased alcohol intake of 210–420 g per week for males or 140–350 g for females. Participants with concomitant LDs were excluded.

^c^Defined as SLD with ≥ 1 cardiometabolic risk factor and excessive alcohol intake (weekly intake > 420 g for males or > 350 g for females) or ALD. Participants with other concomitant LDs were excluded.

^d^Defined as SLD with ≥ 1 cardiometabolic risk factor and other concomitant LDs except for ALD. Participants with excessive alcohol consumption were excluded.

^e^Calculated using the chronic kidney disease epidemiology collaboration equation (2009).

**Table 2:** Association of MASLD and related SLD with outcomes.

	No. of participants	No. of cases (%)	Crude model		Multivariable model 1^b^		Multivariable model 2^c^	
			HR (95% CI)	*p* value^a^	HR (95% CI)	*p* value^a^	HR (95% CI)	*p* value^a^
Liver-related events								
No MASLD/related SLD	226,639	615 (0.27)	Reference	—	Reference	—	Reference	—
MASLD/related SLD	142,247	1480 (1.04)	3.91 (3.56, 4.30)	< 0.0001	3.07 (2.79, 3.39)	< 0.0001	3.00 (2.72, 3.31)	< 0.0001
Pure MASLD^d^	102,821	853 (0.83)	3.11 (2.81, 3.45)	< 0.0001	2.53 (2.27, 2.81)	< 0.0001	2.46 (2.21, 2.73)	< 0.0001
MetALD^e^	28,563	269 (0.94)	3.52 (3.05, 4.07)	< 0.0001	2.81 (2.43, 3.26)	< 0.0001	2.77 (2.39, 3.21)	< 0.0001
MASLD with ALD^f^	10,577	329 (3.11)	12.0 (10.5, 13.7)	< 0.0001	9.05 (7.87, 10.4)	< 0.0001	8.73 (7.59, 10.1)	< 0.0001
MASLD with other etiology^g^	245	21 (8.57)	36.0 (23.3, 55.6)	< 0.0001	27.3 (17.7, 42.3)	< 0.0001	26.5 (17.1, 41.0)	< 0.0001
Liver cirrhosis and cirrhosis-related complications								
No MASLD/related SLD	226,639	551 (0.24)	Reference	—	Reference	—	Reference	—
MASLD/related SLD	142,247	1396 (0.98)	4.12 (3.73, 4.54)	< 0.0001	3.26 (2.94, 3.61)	< 0.0001	3.18 (2.87, 3.52)	< 0.0001
Pure MASLD^d^	102,821	800 (0.78)	3.26 (2.92, 3.63)	< 0.0001	2.66 (2.38, 2.98)	< 0.0001	2.59 (2.31, 2.89)	< 0.0001
MetALD^e^	28,563	250 (0.88)	3.65 (3.15, 4.24)	< 0.0001	2.94 (2.52, 3.43)	< 0.0001	2.90 (2.49, 3.38)	< 0.0001
MASLD with ALD^f^	10,577	321 (3.03)	13.1 (11.4, 15.0)	< 0.0001	9.91 (8.58, 11.5)	< 0.0001	9.54 (8.26, 11.0)	< 0.0001
MASLD with other etiology^g^	245	17 (6.94)	32.5 (20.1, 52.7)	< 0.0001	24.6 (15.1, 39.9)	< 0.0001	23.8 (14.6, 38.6)	< 0.0001
Cancer								
No MASLD/related SLD	226,639	26,186 (11.6)	Reference	—	Reference	—	Reference	—
MASLD/related SLD	142,247	20,511 (14.4)	1.28 (1.26, 1.30)	< 0.0001	1.13 (1.11, 1.15)	< 0.0001	1.12 (1.10, 1.14)	< 0.0001
Pure MASLD^d^	102,821	14,533 (14.1)	1.25 (1.23, 1.28)	< 0.0001	1.11 (1.08, 1.13)	< 0.0001	1.10 (1.08, 1.13)	< 0.0001
MetALD^e^	28,563	4252 (14.9)	1.32 (1.28, 1.36)	< 0.0001	1.15 (1.11, 1.19)	< 0.0001	1.15 (1.11, 1.19)	< 0.0001
MASLD with ALD^f^	10,577	1666 (15.8)	1.42 (1.35, 1.49)	< 0.0001	1.27 (1.20, 1.33)	< 0.0001	1.26 (1.20, 1.33)	< 0.0001
MASLD with other etiology^g^	245	50 (20.4)	1.94 (1.47, 2.57)	< 0.0001	1.78 (1.35, 2.35)	< 0.0001	1.78 (1.35, 2.34)	< 0.0001
Hepatocellular carcinoma								
No MASLD/related SLD	226,639	83 (0.04)	Reference	—	Reference	—	Reference	—
MASLD/related SLD	142,247	206 (0.14)	4.03 (3.12, 5.20)	< 0.0001	2.72 (2.09, 3.54)	< 0.0001	2.73 (2.10, 3.55)	< 0.0001
Pure MASLD^d^	102,821	118 (0.11)	3.19 (2.41, 4.22)	< 0.0001	1.99 (1.49, 2.67)	< 0.0001	2.01 (1.50, 2.69)	< 0.0001
MetALD^e^	28,563	43 (0.15)	4.17 (2.88, 6.02)	< 0.0001	3.54 (2.38, 5.25)	< 0.0001	3.51 (2.36, 5.21)	< 0.0001
MASLD with ALD^f^	10,577	34 (0.32)	9.11 (6.12, 13.6)	< 0.0001	7.40 (4.81, 11.4)	< 0.0001	7.19 (4.67, 11.1)	< 0.0001
MASLD with other etiology^g^	245	9 (3.67)	110.2 (55.4, 219.3)	< 0.0001	75.9 (37.9, 152)	< 0.0001	76.3 (38.1, 152.8)	< 0.0001
Extrahepatic carcinoma								
No MASLD/related SLD	226,639	26,115 (11.5)	Reference	—	Reference	—	Reference	—
MASLD/related SLD	142,247	20,356 (14.3)	1.27 (1.25, 1.30)	< 0.0001	1.12 (1.10, 1.14)	< 0.0001	1.12 (1.10, 1.14)	< 0.0001
Pure MASLD^d^	102,821	14,449 (14.1)	1.25 (1.22, 1.27)	< 0.0001	1.10 (1.08, 1.13)	< 0.0001	1.10 (1.08, 1.12)	< 0.0001
MetALD^e^	28,563	4217 (14.8)	1.31 (1.27, 1.35)	< 0.0001	1.14 (1.11, 1.18)	< 0.0001	1.14 (1.10, 1.18)	< 0.0001
MASLD with ALD^f^	10,577	1638 (15.5)	1.40 (1.33, 1.47)	< 0.0001	1.25 (1.19, 1.31)	< 0.0001	1.24 (1.18, 1.31)	< 0.0001
MASLD with other etiology^g^	245	43 (17.6)	1.66 (1.23, 2.24)	0.0009	1.52 (1.12, 2.04)	0.0065	1.51 (1.12, 2.04)	0.007
All-cause death								
No MASLD/related SLD	226,639	11,809 (5.21)	Reference	—	Reference	—	Reference	—
MASLD/related SLD	142,247	12,703 (8.93)	1.75 (1.70, 1.79)	< 0.0001	1.37 (1.34, 1.41)	< 0.0001	1.32 (1.29, 1.36)	< 0.0001
Pure MASLD^d^	102,821	8978 (8.73)	1.71 (1.66, 1.75)	< 0.0001	1.34 (1.30, 1.38)	< 0.0001	1.28 (1.25, 1.32)	< 0.0001
MetALD^e^	28,563	2402 (8.41)	1.64 (1.57, 1.71)	< 0.0001	1.30 (1.24, 1.36)	< 0.0001	1.27 (1.21, 1.33)	< 0.0001
MASLD with ALD^f^	10,577	1260 (11.91)	2.38 (2.24, 2.52)	< 0.0001	1.90 (1.79, 2.01)	< 0.0001	1.83 (1.72, 1.94)	< 0.0001
MASLD with other etiology^g^	245	50 (20.4)	4.29 (3.25, 5.66)	< 0.0001	3.56 (2.70, 4.71)	< 0.0001	3.46 (2.62, 4.57)	< 0.0001
Liver-related death								
No MASLD/related SLD	226,639	248 (0.11)	Reference	—	Reference	—	Reference	—
MASLD/related SLD	142,247	528 (0.37)	3.46 (2.97, 4.02)	< 0.0001	2.73 (2.34, 3.20)	< 0.0001	2.72 (2.32, 3.18)	< 0.0001
Pure MASLD^d^	102,821	272 (0.26)	2.46 (2.07, 2.92)	< 0.0001	2.01 (1.68, 2.40)	< 0.0001	1.99 (1.67, 2.38)	< 0.0001
MetALD^e^	28,563	116 (0.41)	3.76 (3.02, 4.69)	< 0.0001	3.08 (2.45, 3.87)	< 0.0001	3.06 (2.43, 3.84)	< 0.0001
MASLD with ALD^f^	10,577	129 (1.22)	11.6 (9.36, 14.3)	< 0.0001	9.23 (7.37, 11.6)	< 0.0001	8.92 (7.12, 11.2)	< 0.0001
MASLD with other etiology^g^	245	9 (3.67)	36.7 (18.9, 71.4)	< 0.0001	30.0 (15.4, 58.4)	< 0.0001	29.6 (15.2, 57.7)	< 0.0001
Cancer death								
No MASLD/related SLD	226,639	5829 (2.57)	Reference	—	Reference	—	Reference	—
MASLD/related SLD	142,247	5530 (3.89)	1.54 (1.48, 1.60)	< 0.0001	1.30 (1.25, 1.35)	< 0.0001	1.29 (1.24, 1.34)	< 0.0001
Pure MASLD^d^	102,821	3870 (3.76)	1.49 (1.43, 1.55)	< 0.0001	1.26 (1.20, 1.31)	< 0.0001	1.24 (1.19, 1.29)	< 0.0001
MetALD^e^	28,563	1123 (3.93)	1.55 (1.45, 1.65)	< 0.0001	1.31 (1.23, 1.40)	< 0.0001	1.31 (1.22, 1.39)	< 0.0001
MASLD with ALD^f^	10,577	509 (4.81)	1.94 (1.77, 2.12)	< 0.0001	1.69 (1.54, 1.85)	< 0.0001	1.67 (1.52, 1.83)	< 0.0001
MASLD with other etiology^g^	245	24 (9.80)	4.15 (2.78, 6.19)	< 0.0001	3.77 (2.52, 5.63)	< 0.0001	3.73 (2.50, 5.58)	< 0.0001

Abbreviations: ALD = alcohol fatty liver disease; CI = confidential interval; HR = hazard ratio; LD = liver disease; MASLD = metabolic dysfunction–associated steatotic liver disease; MetALD = MASLD with greater alcohol consumption; SLD = steatotic liver diseases.

^a^Analysis by Cox proportional hazards model.

^b^Adjusted for age, sex, ethnicity, education, Townsend deprivation index, income levels, smoking status, alcohol intake, and activity group by the International Physical Activity Questionnaire.

^c^Additionally adjusted for estimated glomerular filtration rate and cardiovascular diseases.

^d^Defined as SLD with ≥ 1 predefined cardiometabolic risk factor. Participants with increased or excessive alcohol intake (weekly intake ≥ 210 g for males or ≥ 140 g for females) or concomitant LDs were excluded.

^e^Defined as SLD with ≥ 1 cardiometabolic risk factor and an increased alcohol intake of 210–420 g per week for males or 140–350 g for females. Participants with concomitant LDs were excluded.

^f^Defined as SLD with ≥ 1 cardiometabolic risk factor and excessive alcohol intake (weekly intake > 420 g for males or > 350 g for females) or ALD. Participants with other concomitant LDs were excluded.

^g^Defined as SLD with ≥ 1 cardiometabolic risk factor and other concomitant LDs except for ALD. Participants with excessive alcohol consumption were excluded.

## Data Availability

The data that support the findings of this study are available from the United Kingdom's Biobank Board but restrictions apply to the availability of these data, which were used under license for the current study, and so are not publicly available. Data are, however, available from the authors upon reasonable request and with permission of the United Kingdom's Biobank Board.

## Nomenclature

ALD: Alcohol-related liver disease
BMI: Body mass index
CVD: Cardiovascular diseases
FLI: Fatty liver index
HCC: Hepatocellular carcinoma
IPAQ: International Physical Activity Questionnaire
LD: Liver disease
MAFLD: Metabolic dysfunction–associated fatty liver disease
MASLD: Metabolic dysfunction–associated steatotic liver disease
NAFLD: Nonalcoholic fatty liver disease
SLD: Steatotic liver disease
TDI: Townsend deprivation index

## Supporting Information

Table S1: International Classification of Disease-10 codes for baseline exclusions and outcomes.

Table S2: Association of MAFLD with outcomes.

Table S3: Association of MASLD and related SLD with outcomes, additional adjusted model.

Table S4: Association of MASLD and related SLD or MAFLD with liver-related events excluding participants with incident events within the first 2 years of follow-up.

Table S5: Association of MASLD and related SLD or MAFLD with liver-related events taking into account competing risk of death from other causes.

Table S6: Association of MASLD and related SLD or MAFLD with liver-related events with SLD defined as a FLI ≥ 30.
