# Supplementary material for: Risk Heterogeneity of Liver-Related Events and Extrahepatic Outcomes Across MASLD Phenotypes and Risk Stratification by Liver Fibrosis
Source: Int J Endocrinol. 2025 Jun 1;2025:1262001. doi: 10.1155/ije/1262001 (PMC12145934; doi:10.1155/ije/1262001)
Supplement: Supporting Information — Additional supporting information can be found online in the Supporting Information section. [file 1262001.f1.docx]

**SUPPLEMENTARY MATERIALS**

**Association of metabolic dysfunction-associated steatotic liver disease**

**with osteoarthritis: A Prospective Cohort Study**

***Contents***

[***Supplementary Table 1. International Classification of Disease-10 codes for baseline exclusions and outcomes..***](#_Toc159414247)

[***Supplementary Table 2. Association of MAFLD with outcomes.***](#_Toc159414250)

[***Supplementary Table 3. Association of MASLD and related SLD with outcomes, additional adjusted model.***](#_Toc159414251)

[***Supplementary Table 4. Association of MASLD and related SLD or MAFLD with liver-related events excluding participants with incident events within the first 2 years of follow-up***](#_Toc159414252)

[***Supplementary Table 5. Association of MASLD and related SLD or MAFLD with liver-related events taking account competing risk of death from other causes.***](#_Toc159414253)

[***Supplementary Table 6. Association of MASLD and related SLD or MAFLD with liver-related events with SLD defined as a FLI ≥30***](#_Toc159414254)

| **Table S1. International Classification of Disease-10 codes for baseline exclusions and outcomes.** | | |
| --- | --- | --- |
|  | Data field | International Classification of Disease-10 codes |
| **Baseline exclusions** |  |  |
| Cancer | 41270 | C*, excluding 'C443', 'C449' |
| Liver-related events | 41270 | 'K703', 'K729', 'K746', 'K766', 'K767', 'I850', 'I859', 'I982', 'I983', 'C220' |
| **Outcomes** |  |  |
| Liver-related events | 41270 | 'K703', 'K729', 'K746', 'K766', 'K767', 'I850', 'I859', 'I982', 'I983', 'C220' |
| Liver cirrhosis and cirrhosis-related complications | 41270 | 'K703', 'K729', 'K746', 'K766', 'K767', 'I850', 'I859', 'I982', 'I983' |
| Cancer | 41270 | C*, excluding 'C443', 'C449' |
| Hepatocellular carcinoma | 41270 | 'C220' |
| Extrahepatic carcinoma | 41270 | C*, excluding C22*, 'C443', 'C449' |
| All-cause death | 40000 | - |
| Liver-related death | 40001 | K70*, K71*, K72*, K73*, K74*, K75*, K76*, K77*, 'C220', 'C221', 'C227', 'C228', 'C229' |
| Cancer death | 40001 | C*, excluding 'C443', 'C449' |

| **Table S2. Association of MAFLD with outcomes.** | | | | | | | | |
| --- | --- | --- | --- | --- | --- | --- | --- | --- |
|  | No. of participants | No. of cases (%) | Crude model | | Multivariable model 1 ^b^ | | Multivariable model 2 ^c^ | |
|  |  |  | HR (95% CI) | *P* value ^a^ | HR (95% CI) | *P* value ^a^ | HR (95% CI) | *P* value ^a^ |
| **Liver-related events** |  |  |  |  |  |  |  |  |
| MAFLD- | 227006 | 629 (0.28) | Reference | - | Reference | - | Reference | - |
| MAFLD+ | 141880 | 1466 (1.03) | 3.80 (3.46, 4.18) | <0.0001 | 2.98 (2.71, 3.29) | <0.0001 | 2.91 (2.64, 3.21) | <0.0001 |
| **Liver cirrhosis and cirrhosis-related complications** |  |  |  |  |  |  |  |  |
| MAFLD- | 227006 | 565 (0.25) | Reference | - | Reference | - | Reference | - |
| MAFLD+ | 141880 | 1382 (0.97) | 3.99 (3.62, 4.40) | <0.0001 | 3.15 (2.85, 3.49) | <0.0001 | 3.07 (2.78, 3.40) | <0.0001 |
| **Cancer** |  |  |  |  |  |  |  |  |
| MAFLD- | 227006 | 26227 (11.6) | Reference | - | Reference | - | Reference | - |
| MAFLD+ | 141880 | 20470 (14.4) | 1.28 (1.26, 1.30) | <0.0001 | 1.13 (1.11, 1.15) | <0.0001 | 1.12 (1.10, 1.15) | <0.0001 |
| **Hepatocellular carcinoma** |  |  |  |  |  |  |  |  |
| MAFLD- | 227006 | 83 (0.04) | Reference | - | Reference | - | Reference | - |
| MAFLD+ | 141880 | 206 (0.15) | 4.04 (3.13, 5.22) | <0.0001 | 2.73 (2.10, 3.55) | <0.0001 | 2.74 (2.11, 3.57) | <0.0001 |
| **Extrahepatic carcinoma** |  |  |  |  |  |  |  |  |
| MAFLD- | 227006 | 26156 (11.5) | Reference | - | Reference | - | Reference | - |
| MAFLD+ | 141880 | 20315 (14.3) | 1.27 (1.25, 1.30) | <0.0001 | 1.12 (1.10, 1.14) | <0.0001 | 1.12 (1.10, 1.14) | <0.0001 |
| **All-cause death** |  |  |  |  |  |  |  |  |
| MAFLD- | 227006 | 11838 (5.21) | Reference | - | Reference | - | Reference | - |
| MAFLD+ | 141880 | 12674 (8.93) | 1.75 (1.70, 1.79) | <0.0001 | 1.37 (1.34, 1.41) | <0.0001 | 1.32 (1.29, 1.35) | <0.0001 |
| **Liver-related death** |  |  |  |  |  |  |  |  |
| MAFLD- | 227006 | 256 (0.11) | Reference | - | Reference | - | Reference | - |
| MAFLD+ | 141880 | 520 (0.37) | 3.31 (2.85, 3.85) | <0.0001 | 2.61 (2.24, 3.05) | <0.0001 | 2.59 (2.22, 3.03) | <0.0001 |
| **Cancer death** |  |  |  |  |  |  |  |  |
| MAFLD- | 227006 | 5838 (2.57) | Reference | - | Reference | - | Reference | - |
| MAFLD+ | 141880 | 5521 (3.89) | 1.54 (1.48, 1.60) | <0.0001 | 1.30 (1.25, 1.35) | <0.0001 | 1.29 (1.24, 1.34) | <0.0001 |
| CI, confidential interval; HR, hazard ratio; MAFLD, metabolic dysfunction associated fatty liver disease. | | | | | | | | |
| ^a^Analysis by Cox proportional hazards model. | | | | | | | | |
| ^b^Adjusted for age, sex, ethnicity, education, Townsend deprivation index, income levels, smoking status, alcohol intake and activity group by the International Physical Activity Questionnaire. | | | | | | | | |
| ^c^Additionally adjusted for estimated glomerular filtration rate and cardiovascular diseases. | | | | | | | | |

| **Table S3. Association of MASLD and related SLD with outcomes, additional adjusted model ^a^.** | | | |
| --- | --- | --- | --- |
|  |  | HR (95% CI) | *P* value |
| **Liver-related events** | No MASLD/related SLD | Reference | - |
|  | Pure MASLD^b^ | 2.47 (2.13, 2.85) | <0.0001 |
|  | MetALD^c^ | 2.97 (2.50, 3.53) | <0.0001 |
|  | MASLD with ALD^d^ | 9.08 (7.68, 10.7) | <0.0001 |
|  | MASLD with other LDs^e^ | 22.2 (14.2, 34.7) | <0.0001 |
| **Liver cirrhosis and cirrhosis-related complications** | No MASLD/related SLD | Reference | - |
|  | Pure MASLD^b^ | 2.67 (2.29, 3.11) | <0.0001 |
|  | MetALD^c^ | 3.17 (2.64, 3.80) | <0.0001 |
|  | MASLD with ALD^d^ | 10.1 (8.52, 12.1) | <0.0001 |
|  | MASLD with other LDs^e^ | 20.4 (12.4, 33.4) | <0.0001 |
| **Cancer** | No MASLD/related SLD | Reference | - |
|  | Pure MASLD^b^ | 1.09 (1.06, 1.12) | <0.0001 |
|  | MetALD^c^ | 1.14 (1.09, 1.18) | <0.0001 |
|  | MASLD with ALD^d^ | 1.25 (1.18, 1.32) | <0.0001 |
|  | MASLD with other LDs^e^ | 1.73 (1.31, 2.29) | 0.0001 |
| **Hepatocellular carcinoma** | No MASLD/related SLD | Reference | - |
|  | Pure MASLD^b^ | 1.54 (1.05, 2.27) | 0.0293 |
|  | MetALD^c^ | 2.84 (1.78, 4.54) | <0.0001 |
|  | MASLD with ALD^d^ | 5.61 (3.40, 9.24) | <0.0001 |
|  | MASLD with other LDs^e^ | 48.1 (23.0, 100.3) | <0.0001 |
| **Extrahepatic carcinoma** | No MASLD/related SLD | Reference | - |
|  | Pure MASLD^b^ | 1.09 (1.06, 1.12) | <0.0001 |
|  | MetALD^c^ | 1.13 (1.09, 1.18) | <0.0001 |
|  | MASLD with ALD^d^ | 1.23 (1.16, 1.30) | <0.0001 |
|  | MASLD with other LDs^e^ | 1.48 (1.09, 1.99) | 0.0109 |
| **All-cause death** | No MASLD/related SLD | Reference | - |
|  | Pure MASLD^b^ | 1.23 (1.18, 1.28) | <0.0001 |
|  | MetALD^c^ | 1.25 (1.19, 1.32) | <0.0001 |
|  | MASLD with ALD^d^ | 1.77 (1.66, 1.89) | <0.0001 |
|  | MASLD with other LDs^e^ | 3.04 (2.30, 4.02) | <0.0001 |
| **Liver-related death** | No MASLD/related SLD | Reference | - |
|  | Pure MASLD^b^ | 2.23 (1.76, 2.84) | <0.0001 |
|  | MetALD^c^ | 3.65 (2.77, 4.80) | <0.0001 |
|  | MASLD with ALD^d^ | 10.4 (7.90, 13.6) | <0.0001 |
|  | MASLD with other LDs^e^ | 28.4 (14.3, 56.3) | <0.0001 |
| **Cancer death** | No MASLD/related SLD | Reference | - |
|  | Pure MASLD^b^ | 1.21 (1.14, 1.28) | <0.0001 |
|  | MetALD^c^ | 1.29 (1.20, 1.39) | <0.0001 |
|  | MASLD with ALD^d^ | 1.64 (1.48, 1.81) | <0.0001 |
|  | MASLD with other LDs^e^ | 3.51 (2.35, 5.26) | <0.0001 |
| ALD, alcohol fatty liver disease; CI, confidential interval; HR, hazard ratio; LD, liver disease; MASLD, metabolic dysfunction-associated steatotic liver disease; MetALD, MASLD with greater alcohol consumption; SLD, steatotic liver diseases. | | | |
| ^a^Cox proportional hazards model, adjusted for age, sex, ethnicity, education, Townsend deprivation index, income levels, smoking status, alcohol intake, activity group by the International Physical Activity Questionnaire, estimated glomerular filtration rate, cardiovascular diseases, body mass index, diabetes, hypertension, and hyperlipidemia. | | | |
| ^b^Defined as SLD with ≥1 pre-defined cardiometabolic risk factor. Participants with increased or excessive alcohol intake (weekly intake ≥210g for males or ≥140g for females) or concomitant LDs were excluded. | | | |
| ^c^Defined as SLD with ≥1 cardiometabolic risk factor and an increased alcohol intake of 210-420g per week for males or 140-350g for females. Participants with concomitant LDs were excluded. | | | |
| ^d^Defined as SLD with ≥1 cardiometabolic risk factor and excessive alcohol intake (weekly intake >420g for males or >350g for females) or ALD. Participants with other concomitant LDs were excluded. | | | |
| ^e^Defined as SLD with ≥1 cardiometabolic risk factor and other concomitant LDs except for ALD. Participants with excessive alcohol consumption were excluded. | | | |

| **Table S4. Association of MASLD and related SLD or MAFLD with liver-related events excluding participants with incident events within the first 2 years of follow-up.** | | | | | | | | |
| --- | --- | --- | --- | --- | --- | --- | --- | --- |
|  | No. of participants | No. of cases (%) | Crude model | | Multivariable model 1 ^b^ | | Multivariable model 2 ^c^ | |
|  |  |  | HR (95% CI) | *P* value ^a^ | HR (95% CI) | *P* value ^a^ | HR (95% CI) | *P* value ^a^ |
| **MASLD and related SLD** |  |  |  |  |  |  |  |  |
| No MASLD/related SLD | 226588 | 564 (0.25) | Reference | - | Reference | - | Reference | - |
| Pure MASLD^d^ | 102769 | 801 (0.78) | 3.19 (2.87, 3.56) | <0.0001 | 2.60 (2.33, 2.90) | <0.0001 | 2.52 (2.26, 2.82) | <0.0001 |
| MetALD^e^ | 28549 | 255 (0.89) | 3.65 (3.15, 4.23) | <0.0001 | 2.93 (2.52, 3.41) | <0.0001 | 2.89 (2.48, 3.37) | <0.0001 |
| MASLD with ALD^f^ | 10540 | 292 (2.77) | 11.6 (10.1, 13.4) | <0.0001 | 8.90 (7.67, 10.3) | <0.0001 | 8.60 (7.42, 10.0) | <0.0001 |
| MASLD with other etiology^g^ | 239 | 15 (6.28) | 28.3 (17.0, 47.3) | <0.0001 | 21.8 (13.0, 36.5) | <0.0001 | 21.1 (12.6, 35.3) | <0.0001 |
|  |  |  |  |  |  |  |  |  |
| **MAFLD** |  |  |  |  |  |  |  |  |
| MAFLD- | 226952 | 575 (0.25) | Reference | - | Reference | - | Reference | - |
| MAFLD+ | 141773 | 1359 (0.96) | 3.86 (3.50, 4.26) | <0.0001 | 3.05 (2.76, 3.37) | <0.0001 | 2.98 (2.69, 3.29) | <0.0001 |
| ALD, alcohol fatty liver disease; CI, confidential interval; HR, hazard ratio; LD, liver disease; MAFLD, metabolic dysfunction associated fatty liver disease; MASLD, metabolic dysfunction-associated steatotic liver disease; MetALD, MASLD with greater alcohol consumption; SLD, steatotic liver diseases. | | | | | | | | |
| ^a^Analysis by Cox proportional hazards model. | | | | | | | | |
| ^b^Adjusted for age, sex, ethnicity, education, Townsend deprivation index, income levels, smoking status, alcohol intake and activity group by the International Physical Activity Questionnaire. | | | | | | | | |
| ^c^Additionally adjusted for estimated glomerular filtration rate and cardiovascular diseases. | | | | | | | | |
| ^d^Defined as SLD with ≥1 pre-defined cardiometabolic risk factor. Participants with increased or excessive alcohol intake (weekly intake ≥210g for males or ≥140g for females) or concomitant LDs were excluded. | | | | | | | | |
| ^e^Defined as SLD with ≥1 cardiometabolic risk factor and an increased alcohol intake of 210-420g per week for males or 140-350g for females. Participants with concomitant LDs were excluded. | | | | | | | | |
| ^f^Defined as SLD with ≥1 cardiometabolic risk factor and excessive alcohol intake (weekly intake >420g for males or >350g for females) or ALD. Participants with other concomitant LDs were excluded. | | | | | | | | |
| ^g^Defined as SLD with ≥1 cardiometabolic risk factor and other concomitant LDs except for ALD. Participants with excessive alcohol consumption were excluded. | | | | | | | | |

| **Table S5. Association of MASLD and related SLD or MAFLD with liver-related events taking account competing risk of death from other causes.** | | | | | | | | |
| --- | --- | --- | --- | --- | --- | --- | --- | --- |
|  | No. of participants | No. of cases (%) | Crude model | | Multivariable model 1 ^b^ | | Multivariable model 2 ^c^ | |
|  |  |  | HR (95% CI) | *P* value ^a^ | HR (95% CI) | *P* value ^a^ | HR (95% CI) | *P* value ^a^ |
| **MASLD and related SLD** |  |  |  |  |  |  |  |  |
| No MASLD/related SLD | 226639 | 615 (0.27) | Reference | - | Reference | - | Reference | - |
| Pure MASLD^d^ | 102821 | 853 (0.83) | 3.07 (2.76, 3.40) | <0.0001 | 2.51 (2.25, 2.80) | <0.0001 | 2.45 (2.19, 2.73) | <0.0001 |
| MetALD^e^ | 28563 | 269 (0.94) | 3.48 (3.02, 4.02) | <0.0001 | 2.80 (2.41, 3.25) | <0.0001 | 2.77 (2.39, 3.22) | <0.0001 |
| MASLD with ALD^f^ | 10577 | 329 (3.11) | 11.6 (10.2, 13.3) | <0.0001 | 8.84 (7.66, 10.2) | <0.0001 | 8.54 (7.40, 9.87) | <0.0001 |
| MASLD with other etiology^g^ | 245 | 21 (8.57) | 33.8 (21.6, 52.7) | <0.0001 | 25.7 (16.4, 40.4) | <0.0001 | 25.2 (16.0, 39.6) | <0.0001 |
|  |  |  |  |  |  |  |  |  |
| **MAFLD** |  |  |  |  |  |  |  |  |
| MAFLD- | 227006 | 629 (0.28) | Reference | - | Reference | - | Reference | - |
| MAFLD+ | 141880 | 1466 (1.03) | 3.74 (3.41, 4.11) | <0.0001 | 2.96 (2.68, 3.27) | <0.0001 | 2.90 (2.62, 3.20) | <0.0001 |
| ALD, alcohol fatty liver disease; CI, confidential interval; HR, hazard ratio; LD, liver disease; MAFLD, metabolic dysfunction associated fatty liver disease; MASLD, metabolic dysfunction-associated steatotic liver disease; SLD, steatotic liver diseases. | | | | | | | | |
| ^a^Analysis by Cox proportional hazards model. | | | | | | | | |
| ^b^Adjusted for age, sex, ethnicity, education, Townsend deprivation index, income levels, smoking status, alcohol intake and activity group by the International Physical Activity Questionnaire. | | | | | | | | |
| ^c^Additionally adjusted for estimated glomerular filtration rate and cardiovascular diseases. | | | | | | | | |
| ^d^Defined as SLD with ≥1 pre-defined cardiometabolic risk factor. Participants with increased or excessive alcohol intake (weekly intake ≥210g for males or ≥140g for females) or concomitant LDs were excluded. | | | | | | | | |
| ^e^Defined as SLD with ≥1 cardiometabolic risk factor and an increased alcohol intake of 210-420g per week for males or 140-350g for females. Participants with concomitant LDs were excluded. | | | | | | | | |
| ^f^Defined as SLD with ≥1 cardiometabolic risk factor and excessive alcohol intake (weekly intake >420g for males or >350g for females) or ALD. Participants with other concomitant LDs were excluded. | | | | | | | | |
| ^g^Defined as SLD with ≥1 cardiometabolic risk factor and other concomitant LDs except for ALD. Participants with excessive alcohol consumption were excluded. | | | | | | | | |

| **Table S6. Association of MASLD and related SLD or MAFLD with liver-related events with SLD defined as a FLI ≥30.** | | | | | | | | |
| --- | --- | --- | --- | --- | --- | --- | --- | --- |
|  | No. of participants | No. of cases (%) | Crude model | | Multivariable model 1 ^b^ | | Multivariable model 2 ^c^ | |
|  |  |  | HR (95% CI) | *P* value ^a^ | HR (95% CI) | *P* value ^a^ | HR (95% CI) | *P* value ^a^ |
| **MASLD and related SLD** |  |  |  |  |  |  |  |  |
| No MASLD/related SLD | 132224 | 268 (0.20) | Reference | - | Reference | - | Reference | - |
| Pure MASLD^d^ | 173976 | 1075 (0.62) | 3.10 (2.71, 3.54) | <0.0001 | 2.30 (2.01, 2.64) | <0.0001 | 2.25 (1.96, 2.58) | <0.0001 |
| MetALD^e^ | 47298 | 328 (0.69) | 3.47 (2.95, 4.08) | <0.0001 | 2.57 (2.18, 3.03) | <0.0001 | 2.55 (2.16, 3.01) | <0.0001 |
| MASLD with ALD^f^ | 14950 | 388 (2.60) | 13.4 (11.5, 15.6) | <0.0001 | 9.17 (7.78, 10.8) | <0.0001 | 8.86 (7.52, 10.4) | <0.0001 |
| MASLD with other etiology^g^ | 381 | 27 (7.09) | 39.1 (26.3, 58.0) | <0.0001 | 26.6 (17.9, 39.6) | <0.0001 | 25.8 (17.3, 38.5) | <0.0001 |
|  |  |  |  |  |  |  |  |  |
| **MAFLD** |  |  |  |  |  |  |  |  |
| MAFLD- | 138005 | 309 (0.22) | Reference | - | Reference | - | Reference | - |
| MAFLD+ | 230881 | 1786 (0.77) | 3.52 (3.12, 3.97) | <0.0001 | 2.53 (2.24, 2.87) | <0.0001 | 2.48 (2.19, 2.82) | <0.0001 |
| ALD, alcohol fatty liver disease; CI, confidential interval; FLI, fatty liver index; HR, hazard ratio; LD, liver disease; MAFLD, metabolic dysfunction associated fatty liver disease; MASLD, metabolic dysfunction-associated steatotic liver disease; SLD, steatotic liver diseases. | | | | | | | | |
| ^a^Analysis by Cox proportional hazards model. | | | | | | | | |
| ^b^Adjusted for age, sex, ethnicity, education, Townsend deprivation index, income levels, smoking status, alcohol intake and activity group by the International Physical Activity Questionnaire. | | | | | | | | |
| ^c^Additionally adjusted for estimated glomerular filtration rate and cardiovascular diseases. | | | | | | | | |
| ^d^Defined as SLD with ≥1 pre-defined cardiometabolic risk factor. Participants with increased or excessive alcohol intake (weekly intake ≥210g for males or ≥140g for females) or concomitant LDs were excluded. | | | | | | | | |
| ^e^Defined as SLD with ≥1 cardiometabolic risk factor and an increased alcohol intake of 210-420g per week for males or 140-350g for females. Participants with concomitant LDs were excluded. | | | | | | | | |
| ^f^Defined as SLD with ≥1 cardiometabolic risk factor and excessive alcohol intake (weekly intake >420g for males or >350g for females) or ALD. Participants with other concomitant LDs were excluded. | | | | | | | | |
| ^g^Defined as SLD with ≥1 cardiometabolic risk factor and other concomitant LDs except for ALD. Participants with excessive alcohol consumption were excluded. | | | | | | | | |
